# Evidence for Multiple Subpopulations of Herpesvirus-Latently Infected Cells

**DOI:** 10.1128/mbio.03473-21

**Published:** 2022-01-04

**Authors:** Justin T. Landis, Ryan Tuck, Yue Pan, Carson N. Mosso, Anthony B. Eason, Razia Moorad, J. Stephen Marron, Dirk P. Dittmer

**Affiliations:** a Lineberger Comprehensive Cancer Center, Department of Microbiology and Immunology, The University of North Carolina at Chapel Hill, Chapel Hill, North Carolina, USA; b Department of Biostatistics, Gillings School of Global Public Health, The University of North Carolina at Chapel Hill, Chapel Hill, North Carolina, USA; c Department of Statistics and Operations Research, The University of North Carolina at Chapel Hill, Chapel Hill, North Carolina, USA; Princeton University

**Keywords:** Kaposi’s sarcoma, herpesvirus, KSHV, single cell, single-cell RNA-seq, lymphoma, primary effusion lymphoma, Castleman’s disease, scRNAseq, Kaposi’s sarcoma-associated herpesvirus

## Abstract

Kaposi’s sarcoma-associated herpesvirus (KSHV)-associated primary effusion lymphomas (PEL) are traditionally viewed as homogenous regarding viral transcription and lineage of origin, but so far this contention has not been explored at the single-cell level. Single-cell RNA sequencing of latently infected PEL supports the existence of multiple subpopulations even within a single cell line. At most 1% of the cells showed evidence of near-complete lytic transcription. The majority of cells only expressed the canonical viral latent transcripts: those originating from the latency locus, the viral interferon regulatory factor locus, and the viral lncRNA nut-1/Pan/T1.1; however, a significant fraction of cells showed various degrees of more permissive transcription, and some showed no evidence of KSHV transcripts whatsoever. Levels of viral interleukin-6 (IL-6)/K2 mRNA emerged as the most distinguishing feature to subset KSHV-infected PEL. One newly uncovered phenotype is the existence of BCBL-1 cells that readily adhered to fibronectin and that displayed mesenchymal lineage-like characteristics.

## INTRODUCTION

Latency is the default outcome after infection with Kaposi’s sarcoma-associated herpesvirus (KSHV) or human herpesvirus 8. Epigenetic profiling showed that the viral genome is heavily and rapidly silenced upon uncoating of the capsid in the nucleus of any infected cell (1–6). Very few viral genes are transcribed in infected cells during the latent phase. Chief among the constitutively expressed genes is the latency-associated nuclear antigen (LANA), which is encoded by open reading frame (ORF) 73 (7–10). LANA is necessary and sufficient to replicate the viral genome in synchrony with the cell and to properly partition the viral plasmids in dividing cells (11–15). All KSHV-infected cells are uniquely marked by punctate foci in the nucleus of infected cells that are composed of the LANA protein and viral DNA (16, 17). Since the LANA protein has a half-life of several days, even cells that initiate lytic viral replication remain positive for LANA protein. Moreover, in those cells, LANA transcription is maintained by the second, lytic promoter of LANA (18). The KSHV latency-locus promoter also directs the expression of the viral cycling homolog (vCyc; ORF 72), the ORF 71 protein, which is called vFLIP, and the K12/Kaposin protein as well as all viral microRNAs (19). K12/Kaposin and vCyc mRNAs can also independently initiate at internal promoters (20, 21). The K12/Kaposin mRNA is the most abundant RNA in KSHV-infected cells and can easily be detected by *in situ* hybridization (22). It gives rise to the Kaposin protein variants as well as the microRNA miR-K12-10a, which is embedded within the Kaposin ORF (23).

Genome-wide transcriptional profiling studies using reverse hybridization (24–30), real-time quantitative PCR arrays (31–34), and bulk RNA sequencing (RNAseq) (35, 36) have generated a complete picture of the average transcription pattern of KSHV in Kaposi’s sarcoma tumor lesions, in experimentally infected cells, and in primary effusion lymphoma (PEL) cell lines. These studies uncovered that different KSHV-associated diseases, such as KS, PEL, or multicentric Castleman’s disease (MCD), exhibit different transcription programs, and that even within a disease, such as KS, different primary lesions exhibit widely varying KSHV transcription patterns (20, 33, 34).

PEL cell lines are the workhorses of KSHV research (37–39). Unlike cells derived from KS lesions, cells derived from a primary patient PEL exudate can be adapted to grow in culture indefinitely (40, 41). These long-term propagated PEL cell lines, as well as primary patient-derived PEL explants, reproducibly form tumors in immune-deficient mice (42, 43). Adaptation to culture, however, is not a very efficient process. Few robust PEL cell lines exist, and the most used PEL cell lines have been in continued culture for over 20 years. They are genetically stable but have acquired many genetic alterations in the human host genome (44, 45). In sum, PEL represents a reproducible and robust model to probe KSHV transcription at the single-cell level.

Viral latent protein expression is continuously required for PEL survival (17, 46). Since PEL tumors and cell lines grow in the presence of the KSHV polymerase inhibitors ganciclovir and Foscarnet (trisodium phosphonoformate) (43, 47), KSHV lytic DNA replication and virion formation is not required for PEL survival. Notably, polymerase inhibitor experiments make no claims as to the need for intermittent immediate-early or early protein expression or for late gene transcription that is not strictly dependent on viral DNA polymerase-mediated DNA replication.

BCBL-1 is perhaps the best-characterized PEL-derived cell line in existence. BCBL-1 was initially derived from a patient with AIDS (37, 48). Unlike other PEL, BCBL-1 does not contain Epstein-Barr virus (EBV). The BCBL-1 strain of KSHV is replication competent and infectious (37, 49, 50). The KSHV strain from BCBL-1 cells has been sequenced multiple times, and the BCBL-1 exome, transcriptome, and epigenome have been described in detail (2–4, 24, 31, 35, 36, 51). The BCBL-1 proteome, metabolome, and kinome are known as well (35, 36, 52, 53). For all intents and purposes, BCBL-1 cultures are considered clonal, overwhelmingly in a latent state of the viral life cycle, and stable regarding viral and cellular gene transcription.

Under normal growth conditions, a minority fraction of BCBL-1 reactivates KSHV spontaneously. The frequency of spontaneous reactivation varies depending on time in continuous culture and health of the culture but does not exceed 5%. The fraction of reactivating cells can be increased to 25% when exposed to saturating conditions of chemical inducers, such as phorbol esters, and/or histone deacetylase (HDAC) inhibitors, such as butyrate or valproic acid (6, 54, 55). The fraction of reactivating cells can be increased to over 50% when providing the KSHV Rta/ORF 50 protein in *trans* (56, 57). This suggests (i) that there exists a natural, host cell signaling-dependent mechanism to change viral transcription and (ii) that virus-intrinsic and cell-dependent layers of regulation render each single cell slightly different from its neighbor.

The following experiments use single-cell RNA sequencing (scRNAseq) to test the hypothesis that, prior to chemical induction, all BCBL-1 cells exhibit the same cellular and viral transcription pattern or that, alternatively, there exists one or more subsets of cells that spontaneously initiated immediate-early, early, or late viral transcription.

scRNAseq represents different things to different people. First, scRNAseq enables high-confidence, high-throughput transcription profiling of a limited number of cells, such as those obtained by laser capture microscopy, fluorescence-activated cell sorting (FACS), or microfluidics, as adopted for the 10xGenomics or BD Rhapsody platform. FACS can be tuned to deliver at most one cell per well into a 96-well culture plate, which is the approach that was used here. Second, scRNAseq enables the analysis of complex mixtures of cells, such as those that exist within a primary tumor biopsy specimen of mixed cellularity or within a heterogeneously infected culture of cells. Recently, a number of studies have applied scRNAseq to acute virus infections, such as to human immunodeficiency virus (HIV) (58, 59), influenza virus (60, 61), or flaviviruses (62). scRNAseq has also been applied to the analysis of mixed cell populations that are latently infected with human cytomegalovirus and other herpesviruses, yielding somewhat contradictory results (63–65). Despite this progress, our understanding of the technical and biological intricacies of applying scRNAseq to virally infected cells remains incomplete.

In this study, scRNAseq is used in its original sense. Given a population of cells, which by any other method, including bulk RNAseq, appears homogenous, one can nevertheless identify novel subpopulations of cells based on their individual transcriptional activity. This is an important question, as it can lead to the discovery of cancer stem cells, of drug-resistant or virus-replication susceptible cells, and of novel biomarkers to characterize those subpopulations.

The following report is divided into three parts. Part one describes the pattern of KSHV transcription in latently infected PEL cells at the single-cell level. Two different PEL cell lines, JSC-1 and BCBL-1, could easily be distinguished based on differing viral transcription alone. This finding reaffirms the requirement to always test multiple cell lines and articulates a need to generate additional PEL cell lines to deliver insights of high rigor, robustness, and repeatability. Several viral genes, such as viral IL-6 (vIL6)/ORF K2, were affirmed as frequently transcribed genes in a fraction of cells. Thus, vIL6/ORF K2 mirrored the variable expression seen clinically in KSHV-associated multicentric Castleman’s disease (16, 66, 67). Part two describes biological and technical variability in the experimental designs and how these features affect the interpretation of scRNAseq data for viral infections. scRNAseq data have less than ideal statistical properties (68, 69). The scRNAseq data for viral gene transcription had even worse statistical properties, since viral genes are transcribed at much lower levels than many cellular genes. Hence, it was important to understand nonrelevant sources of experimental noise and to assign explicit confidence levels to the different clusters of cells and genes. Part three describes the cellular profile of PEL by scRNAseq. Lastly, this study also explores a newly appreciated predilection of some BCBL-1 cells, but not of JSC-1 cells, to grow attached to fibronectin on plastic surfaces. This *in vitro* behavior mirrors the clinical findings that describe an extracavitary variant of PEL (70, 71).

## RESULTS

### Viral transcription patterns in latently infected clonal PEL cell lines.

**FIG 1:**
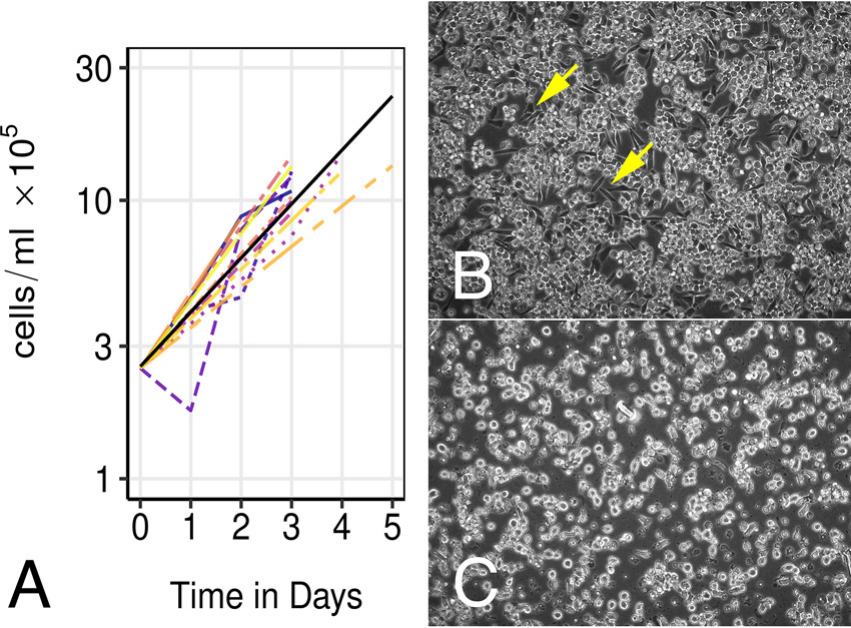
Cell growth characteristics and adhesion phenotype of BCBL1 cells. (A) Shown are growth curves of BCBL-1 at consecutive passages and the linear fit across all the data (black) (*n* = 13 passages). Log_10_ value of cell concentration, in cells per milliliter, is shown on the vertical axis and days postseeding on the horizontal axis. Cells were seeded at 0.25 × 10^6^ cells/ml on day 0. (B and C) Shown is an image (200×) of cultures of either BCBL1 (B) or JSC1 (C) cells that were grown on horizontal fibronectin-coated flasks. The yellow arrows point to some spindle-shaped cells that are flattened out and tightly attached to the fibronectin matrix.

To understand variations in latent KSHV transcription at the single-cell level, viral mRNA levels in PEL were determined by scRNAseq. As scRNAseq is still a relatively new technology, it was important to explicitly explore sources of variability to find robust and repeatable patterns of viral gene transcription (Table 1). Two different PEL cell lines were used, BCBL-1 and JSC-1. To explore differences due to variation within a single cell line, cells were single-cell sorted at three different time points or dates from the same initial seed culture. Each time point was separated by 1 week, i.e., multiple doubling times, from the other. The cells were maintained under ideal culture conditions and collected in mid-log phase for analysis. The experiment was repeated several months later. Growth rates were equivalent across consecutive passages (Fig. 1A), as cells were seeded at the exact same density of 0.25 × 10^6^ cells/ml at day zero and passaged before reaching stationary phase around day five.

**TABLE 1:** Summary of experiments^*a*^

Plate (*N* = 10), ε_1_	Data (*N* = 12)	Date (yr-mo-day) (*N* = 5), ε_2_	Origin (*N* = 2), ε_3_	Cell (*N* = 2), ε_4_	Wells (*N* = 960), ε_5_	Adherence (*N* = 2), ε_6_
1	a	2018-09-04	BCBL1	One	96	No
2	b	2018-09-11	BCBL1	One	96	No
3	c	2018-09-26	BCBL1	One	96	No
4	d	2018-09-26	BCBL1	One	96	No
5	e	2018-09-26	BCBL1	One	96	No
	f	2018-09-26	BCBL1	One	0	No
6	g	2018-09-30	JSC1	One	96	No
	h	2018-09-30	JSC1	One	0	No
7	i	2018-09-30	BCBL1	One	96	Yes
8	j	2018-09-30	BCBL1	Two	96	Yes
9	k	2019-08-08	BCBL1	One	96	Yes
10	l	2019-08-08	BCBL1	One	96	Yes

a The cells were sorted into 10 individual 96-well plates (wells and plate columns) at 5 different dates (date column), each representing a biological replicate or different populations of cells of the same monoclonal origin (origin column). For plate 8, two cells (cell column) were sorted into each well. For plates 7 to 10, BCBL-1 cells were allowed to adhere to plastic and nonadherent cells washed off. ε refers to the factor that was used to normalize across the experiment.

Two types of culture conditions were compared. BCBL-1 cells were either grown in suspension in upright flasks or attached to fibronectin-coated plastic in horizontal flasks (Fig. 1B and C; see also Movie S1 in the supplemental material). Individual cells were sorted into individual wells of a 96-well plate, and libraries were prepared and subjected to scRNAseq. For two biological replicates, the same library was sequenced on two different Illumina lanes. This served as a technical repeat to estimate sequencing accuracy and library depth effect. Each batch of cells produced a comparable number of reads and unique molecular indices (UMI) (Table 2), attesting to the robustness and reproducibility of this method. In addition to reads mapping to the human genome, reads mapping to the KSHV genome were recovered across all samples.

**TABLE 2:** Summary of next-generation sequencing^*a*^

Name	Cell (no. of reads^*b*^)	Cell (no. of UMI)	Cell (no. of genes)	Virus (no. of reads)	Virus (no. of genes)
Plate 1	47,898,415	1,686,753	11,627	737	63
Plate 2	42,308,862	1,051,764	10,774	766	58
Plate 3	70,460,719	1,784,666	11,762	1,917	72
Plate 4	61,547,640	1,854,635	11,980	1,933	73
Plate 5a^*c*^	66,497,202	1,495,992	11,631	1,682	75
Plate 5b^*c*^	61,378,574	1,561,116	11,678	1,829	75
Plate 6a^*c*^	42,925,808	1,685,717	11,933	10,217	70
Plate 6b^*c*^	56,591,011	1,848,909	12,027	11,443	74
Plate 7	97,327,587	3,462,436	13,105	50,485	78
Plate 8	86,963,518	2,457,892	12,403	3,377	77
Plate 9	47,168,617	2,604,976	14,449	1,218	72
Plate 10	57,609,160	1,361,365	10,518	1,116	73
Median ± SEM	6 × 10^7^ ± 5 × 10^6^	1.9 × 10^6^ ± 1.8 × 10^5^	11,847 ± 296	1,873 ± 4,070	73 ± 2

a One Illumina library was prepared for each plate. For plates 5 and 6, the same library was sequenced twice, indicated by a and b. Shown is the total number of reads mapping to either the cell or virus genome, the number of UMIs, and the number of genes detected by at least one read.

b Data are from 2 × 250 paired reads at 3 libraries per lane on an Illumina HiSeq instrument (GenWiz Inc., Plainfield, NJ).

c Data sets a and b were combined for data analysis unless indicated otherwise.

10.1128/mbio.03473-21.1 MOVIE S1 Time-dependent adhesion of BCBL-1 cells to fibronectin-coated plastic. BCBL-1 cells were seeded at 2.5 × 10^5^ cells/mL, plated in 5 mL in a 6-well plate coated with fibronectin in RPMI plus 20% fetal bovine serum, penicillin-streptomycin, l-glutamine, and sodium pyruvate 96-h time lapse (4 days). Download Movie S1, MOV file, 17.5 MB. Copyright © 2022 Landis et al. 2022 Landis et al. https://creativecommons.org/licenses/by/4.0/ This content is distributed under the terms of the Creative Commons Attribution 4.0 International license.

**FIG 2:**
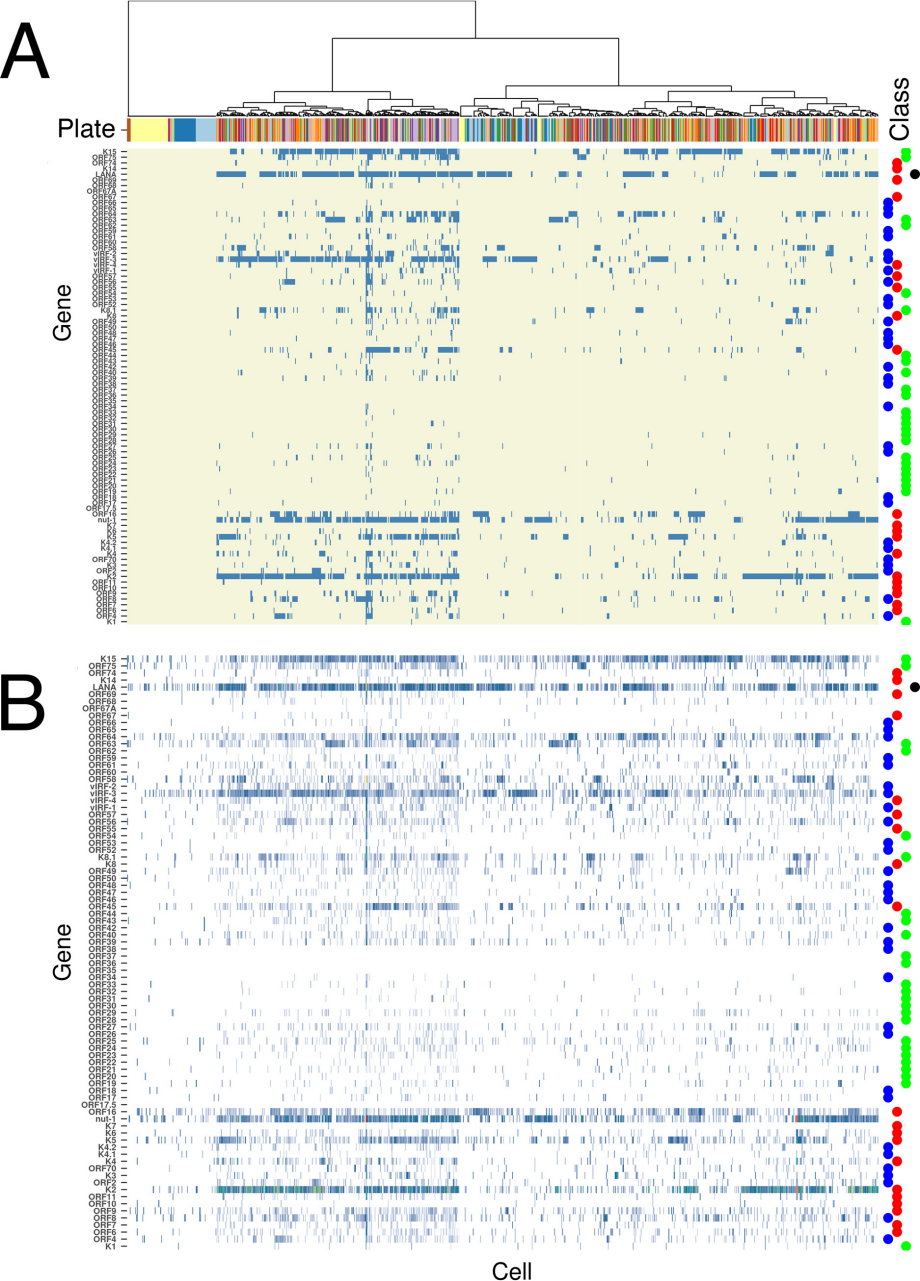
Heatmap of KSHV transcription in PEL. Shown are the results of unsupervised hierarchical clustering of PEL cells (columns) by KSHV genes (rows). Genes are arranged in order from 5′ (bottom) to 3′ (top). A dendrogram on top represents the clustering results, which were based on the binary matrix of detected/undetected genes. A gene was detected if it had at least 2 UMI counts and undetected otherwise. The top heatmap represents this binary signal, whereby detected genes are labeled in blue and nondetected genes are labeled in yellow. The lower heatmap represents the KSHV transcription pattern after log normalization. With this, we can visualize how many genes possessed exactly 1 count.

Next-generation sequencing reads were mapped to the KSHV reference genome NM_009333 and counts per open reading frame (ORF) computed. The KSHV viral miRNA loci were removed prior to analysis, as these are not captured in the cDNA-generating step. The viral latent genes K12, vFLIP, vCyc, and LANA were combined, since transcripts across the latency locus are 3′ coterminal and the scRNAseq assay uses poly(dT) in the initial priming step. Only cells that passed quality control (QC), based on the analysis of cellular transcripts, were used for subsequent detailed analyses. Most of the cells that failed QC and are not represented in Fig. 2 had no detectable KSHV transcripts. As the same cells also had less than the minimum permissible number of cellular mRNAs detected, these most likely represent technical failures.

The LANA/Kaposin/latency locus and nut-1/PAN RNAs were detected even in some of the cells that failed QC. These are the most abundant transcripts in any KSHV-infected cell. Some of the cells that failed QC expressed only the latency transcripts/Kaposin, some only nut-1/PAN, and some both, suggesting that these RNAs were more abundant than any other mRNA. If each cell *a priori* contains both at equivalent levels, this combination pattern (not A and not B, only A, only B, and A and B) is consistent with random fluctuations at the limit of detection for this assay, as would be expected for cells that failed QC precisely because too few transcripts overall were recorded. Conversely, we submit that KSHV mRNA signals in cells that passed QC were not limited by sensitivity and reflect biological fluctuations. Suboptimal library depth was not the reason for differences in KSHV mRNA levels in those cells that passed QC, since sequencing the same library twice (Table 1, a and b replicates) did not change the analysis.

Figure 2 shows a heatmap representation of KSHV gene transcription for each KSHV gene and each cell in the experiment after unsupervised hierarchical clustering. A total of *n* = 841 cells were analyzed across *m* = 84 viral genes. The viral genes are listed in order of their genome location and prior classification into latent, immediate-early, early, and late genes, indicated by colors on the right and the different plates corresponding to the various technical and biological replicates colored on top. Figure 2A shows a binary representation requiring at least two reads to map to an ORF to be marked as detected (blue). Figure 2B shows the log-transformed counts. Thus, the process shown in Fig. 2A maximizes specificity as it filters out genes with only one mapped read, which could be due to misalignment, contamination, or transcriptional noise. This analysis led to the following conclusions.

(i) The most consistently detected mRNAs were nut-1/Pan/T1.1. This was followed by the latency mRNAs mapping to ORF K12, vFLIP, vCYC, and LANA and the vIRF locus, including vIRF1, vIRF2, vIRF3 (LANA-2), and vIRF4. Some cells transcribed nut-1/PAN but not the latency transcripts, others transcribed the latency transcripts but not nut-1/PAN, and others transcribed both. Thus, the scRNAseq broadly confirmed the known transcription profiles of the previously identified viral latent genes in the majority of PEL cells in both cell lines.
(ii) Less than 1% of cells transcribed all viral transcripts or significant amounts of late viral genes. This confirms that under optimal culture conditions, PEL are tightly latent. External stimuli are required to induce lytic reactivation and genome replication. Maintaining PEL under ideal growth conditions (Fig. 1) minimized the spontaneous reactivation rate to yield a lower limit of spontaneous reactivation.
(iii) In some cells (plate 9 [yellow] and, to a lesser degree, plates 1 and 2), no KSHV transcription could be detected at all. In those cells, the entire KSHV genome was transcriptionally silent, or rather the majority of KSHV plasmids within a single cell were inactive even across the LANA promoter (1). This is an unexpected finding, as to date no one has been able to physically isolate a stable subclone of any PEL cell line that is devoid of the KSHV plasmid. However, this finding does correlate with *in situ* studies of LANA (11, 17, 72), which reported variability in the number of LANA dots, a surrogate for the presence of KSHV and LANA transcription per cell. Thus, while not stable over time and subject to extreme negative selection, it seems as if into every generation a transcriptionally silent cell is born, one cell in each population.
(iv) A significant fraction of cells transcribed vIL6/K2 or ORF 2/vDHFR. vIL6 and ORF2/vDHFR are 3′ coterminal and colinear across the entire ORF of vIL6/ORF K2. Hence, this assay could not discern which transcript was measured. vIL6/ORF K2 was the discerning factor between the JSC-1 and BCBL-1 cell lines. Most JSC-1 cells transcribed ORF K2/vIL6. By comparison, few BCBL-1 cells did. It is well established that a subpopulation of PEL, MCD, and KS express ORF K2/vIL6 but also that ORF K2/vIL6 expression is heterogenous and can be regulated by cellular signaling pathways independently of KSHV lytic replication (73, 74). The ORF K2/vIL6 was among the most differentially regulated viral mRNAs among KSHV latently infected B cells.

**FIG 3:**
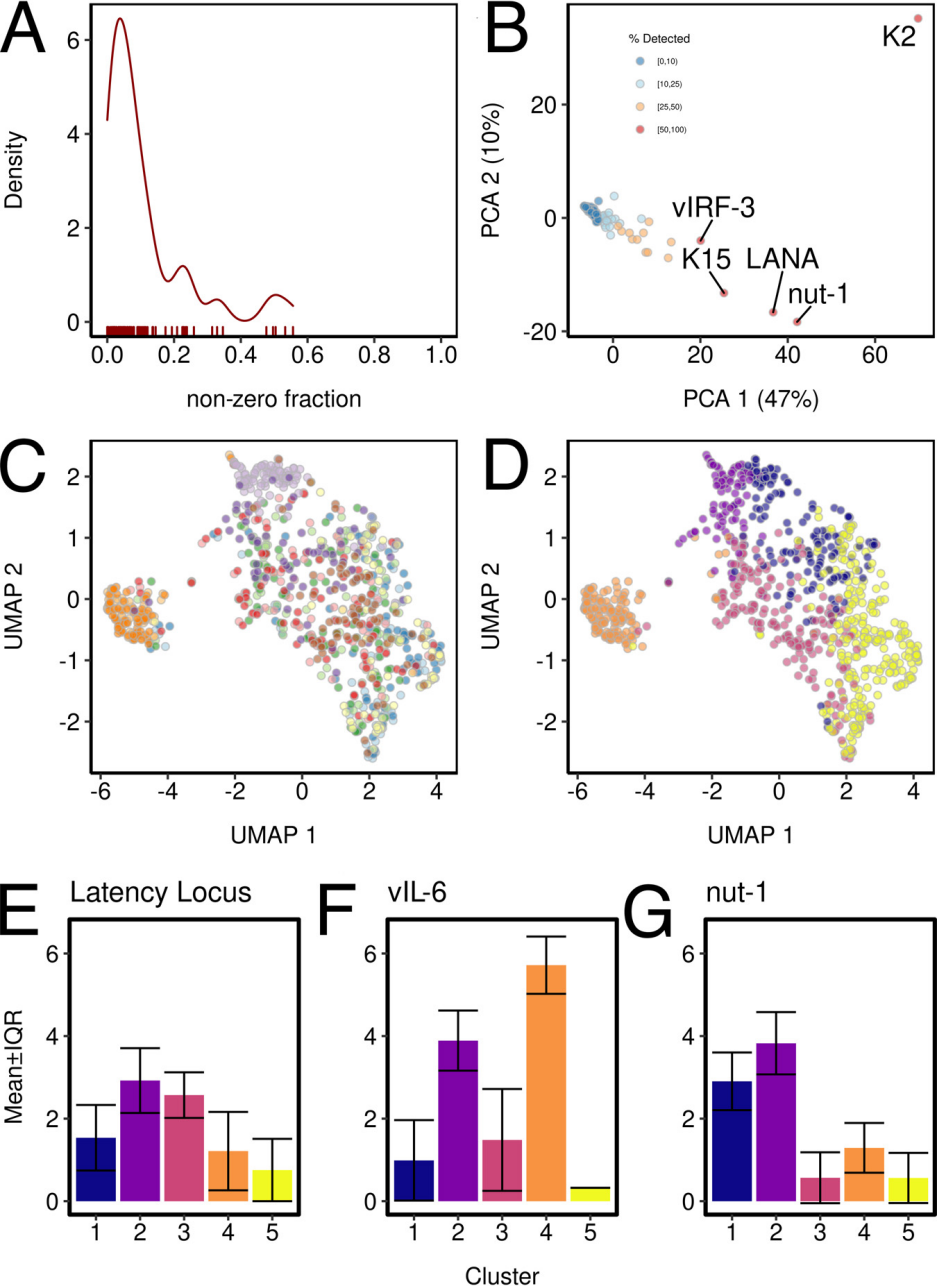
KSHV-based clustering of PEL. (A) Distribution of the detection rates (nonzero fraction) on the horizontal axis. This is a measure of the zero inflation for each gene. (B) Principal-component analysis (PCA) of KSHV genes. LANA, nut-1, and the vIRF have the most distinct expression patterns, and LANA and nut-1 were the most abundantly expressed genes (PC1). (C and D) The same UMAP plot of all cells. In panel C, the cells are color coded by the plate/library number (plate 1, light blue; plate 2, dark blue; plate 3, light green; plate 4, dark green; plate 5A, light red; plate 5B, dark red; plate 6A, light orange; plate 6B, dark orange; plate 7, light purple; plate 8, dark purple; plate 9, yellow; plate 10, brown), and in panel D the cells are color coded by the cluster designation as identified by unsupervised analysis using K-medoids. Cluster identification and per-plate saturation can be found in Table 3. (E) Mean ± interquartile range (IQR)-adjusted counts for the latency locus in each of the clusters, i.e., the relative expression of LANA and the same for vIL-6 (F) and nut-1 (G).

To obtain an unbiased impression of the population structure of the experiment, each cell was assigned to one cluster with related viral gene expression (unsupervised clustering). Each gene was considered following a negative binomial distribution (Fig. 3A). Figure 3B shows a visualization using principal-component analysis (PCA) for the viral genes. Mirroring the raw data, these fall into two groups: those with broad expression across many cells, which includes nut-1, LANA, vIL6/K2, K15, and vIRF-3, each of which are separate from the others, and the remainder of the genes that have a more sporadic expression pattern particular to individual cells, cell lines, or growth conditions. Together, these 5 transcripts are responsible for 47% of the variation in the data (PC1).

The uniform manifold approximation and projection (UMAP) representation of cells’ clusters are not enriched for particular plates or dates (Fig. 3C); rather, they represent cells of similar viral transcription patterns (Fig. 3D). The exception is plate 6, which represents JSC-1 cells only, whereas all other plates represent BCBL-1 cells. Hence, in this case the plate/batch separation mirrors the viral transcription clusters.

As a result of unsupervised clustering, each cell in the experiment was assigned to a cluster representing a specific, composite pattern of KSHV transcription (Table 3). For instance, plates 9 and 10 were enriched for cells with a gene expression pattern identified by clusters 1 and 5 but entirely devoid of cells with a gene expression pattern indicative of clusters 2 and 4. Plate 6 showed the JSC-1 cell-specific pattern of viral transcription.

**TABLE 3:** Viral transcription patterns in latently infected clonal PEL cell lines^*a*^

Plate	Cell line	Value (%) for cluster no.^*b*^				
		1	2	3	4	5
1	BCBL-1	1 (*)	0 (*)	12 (16)	5 (*)	**56 (76)**
2	BCBL-1	9 (11)	3 (*)	18 (23)	3 (*)	**47 (59)**
3	BCBL-1	15 (20)	5 (*)	**29 (39)**	2 (*)	24 (32)
4	BCBL-1	8 (11)	6 (*)	**29 (42)**	3 (*)	23 (33)
5A	BCBL-1	11 (16)	6 (*)	**34 (48)**	4 (*)	16 (23)
5B	BCBL-1	12 (17)	8 (11)	**33 (47)**	3 (*)	15 (21)
6A	JSC-1	0 (*)	1 (*)	0 (*)	**56 (98)**	0 (*)
6B	JSC-1	0 (*)	1 (*)	0 (*)	**57 (98)**	0 (*)
7	BCBL-1	**39 (45)**	**46 (54)**	0 (*)	0 (*)	1 (*)
8	BCBL-1	25 (39)	13 (21)	16 (25)	3 (*)	6 (10)
9	BCBL-1	6 (*)	0 (*)	2 (*)	1 (*)	**60 (89)**
10	BCBL-1	25 (38)	1 (*)	19 (28)	0 (*)	23 (34)

a Shown are the numbers of cells for each plate classified into each unsupervised k-means cluster. Each entry also contains an additional value indicating the percentage of cells within a plate that was classified to a given cluster.

b Shown in parentheses is the percentage of cells or an asterisk if less than 10% of cells are in a particular cluster. Indicated in boldface are the most abundant contributors to each cluster.

Each of these clusters was defined by a KSHV gene signature, rarely a single gene. For instance, clusters 2 and 3 contain the cells with the highest numbers of latency transcripts (Fig. 3E). Clusters 2 and 4 show elevated levels of vIL6/ORF K2 (Fig. 3F) relative to the cells in all other clusters. Clusters 1 and 2 show high levels of nut-1/PAN (Fig. 3G). The clusters seem to be identified with the following profiles: clusters 3, 4, and 1 are high in latency locus, vIL6, and nut-1, respectively, cluster 2 is high in all the aforementioned genes, and cluster 5 is not enriched in any of the genes. The example of vIL6/ORF K2 contains an interesting lesson. Overall, JSC-1 expresses much more vIL6/ORF K2 than BCBL-1 (cluster 4). Hence, experiments aimed at understanding the role of vIL6 in PEL pathogenesis will give different results depending on which cell line is used, as would agents that target IL-6, IL-6 receptor, or IL-6-dependent signaling. However, in each BCBL-1 population there exists a sizable fraction of cells (cluster 2) that transcribe high levels of vIL6/ORF K2. Hence, there would be a signal in BCBL-1 if agents, which target vIL6, IL-6 receptor, or IL-6-dependent signaling, were tested in BCBL-1. Conversely, this suggests that independence from vIL-6 signaling can arise rapidly in PEL, as many cells do fine without vIL6/ORF K2.

This analysis provided the class assignments, also called cluster bins, used in the last part of the manuscript to test the hypothesis that the different patterns of KSHV transcription within the same cell culture flask correlate with different patterns or pathways of host gene transcription.

### The cellular single-cell transcriptome of PEL.

As scRNAseq is still developing as a technology, it was important to determine the experimental factors that contribute to data variability. The overall goal was to report the most robust, reproducible, and specific results that could be garnered from this experimental data. The experimental setup reflected these goals (Table 1) and allowed for detailed analyses using pairwise comparisons. The details of these validation studies are described in Materials and Methods and Fig. S2. These studies established robust, reproducible, and rigorous boundaries in which to interpret these results. The validation studies on cellular transcripts affirmed conventional wisdom: differences in transcription were greater between two different cell lines than among cells originating from the same clonal population, and biological day-to-day variability was greater than variability due to technical differences in single-cell library preparation or sequencing.

10.1128/mbio.03473-21.3 FIG S2 Sensitivity analysis. Shown are pairwise plots of log-normalized cellular gene mean counts (A to D) and the corresponding MA plots (E to H) that show variation on the vertical and mean expression on the horizontal axis. (A and E) Comparisons of the same library sequenced in two different Illumina lanes from BCBL1 cells. (B and F) Comparison of libraries of either one or two cells per well sorted on the same date from BCBL1 cells. (C and G) Comparison of BCBL1 and JSC1 cells collected on the same date. (D and H) Comparison of libraries from two consecutive, biological replicates of BCBL1 cells. The colors black, grey, orange, and red indicate the absolute difference in log mean. Black indicates the value is between 0 and 0.5, grey indicates between 0.5 and 1, yellow indicates between 1 and 2, and red indicates greater than or equal to 2. Download FIG S2, TIF file, 1.7 MB. Copyright © 2022 Landis et al. 2022 Landis et al. https://creativecommons.org/licenses/by/4.0/ This content is distributed under the terms of the Creative Commons Attribution 4.0 International license.

**FIG 4:**
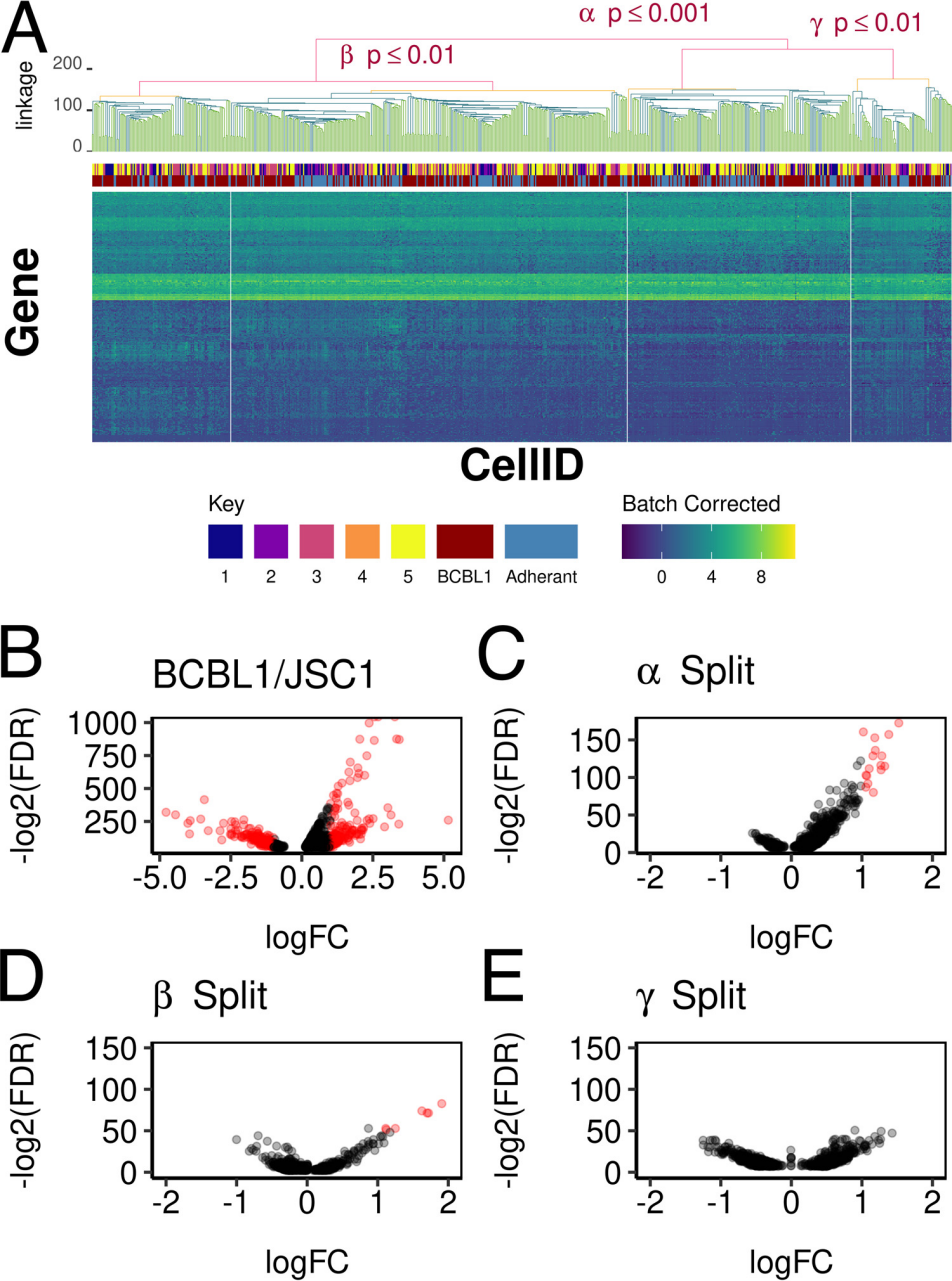
Association between KSHV and host cell transcription. Heatmap of cellular transcription of BCBL-1 cells after batch correction. The associated SigClust dendrogram is above with annotations of which clusters are significant. The KSHV K-means clustering assignments and cell group identity are mapped between the heatmap and the dendrogram. Panel B shows a volcano plot of genes that were differentially regulated between BCBL-1 and JSC-1 cells, panel C shows a volcano plot of genes that signify differences at the major split (*P* ≤ 0.001), panel D shows a volcano plot of the left minor split, and panel E shows a volcano plot of the right minor split (*P* ≤ 0.01). The individual genes are listed in Table S1.

The transcription profile differed significantly between JSC-1 cells and BCBL-1 cells, as shown in Fig. 3 by UMAP analysis. Hence, BCBL-1 cells were analyzed separately to find heterogeneity within a single cell line. This data set encompasses different biological replicates, including culture enriched for adherent cells. Figure 4A shows the result of hierarchical clustering for BCBL-1 cells based on cellular transcript levels. Below the dendrogram are indicated the clusters as determined by viral gene expression and the types of biologically distinct cell types: BCBL-1 floating or BCBL-1 adhering to plastic. Note, however, that this experimental approach represents a rather crude method to select these two states or populations of BCBL-1 cells. Four clusters were identified based on cellular transcription alone and were statistically significant, with a *P* value of ≤0.05 after adjustment for false discovery rate (FDR). These “cellular” clusters did not correlate with viral transcription clusters or the adherent/suspension enrichment status of the BCBL-1 cells.

10.1128/mbio.03473-21.5 TABLE S1 List of top 1,000 cellular genes that changed in Fig. 4 with individual *P* values and mean fold change. The format is .csv. The comparison column indicates which comparison we are performing, and the class column indicates if the dot was red, i.e., having an abs(log(FC)) > 1 and a log_2_(FDR) > 50. Download Table S1, XLSX file, 0.3 MB. Copyright © 2022 Landis et al. 2022 Landis et al. https://creativecommons.org/licenses/by/4.0/ This content is distributed under the terms of the Creative Commons Attribution 4.0 International license.

To identify genes that defined these clusters, a pairwise comparison was performed at each significant hierarchical split. As a positive control, Fig. 4B shows the result for genes that differed between JSC-1 and BCBL-1 cells in the form of a volcano plot. As expected, many transcripts were dramatically different between two cell lines. Note the scales for the fold change on the horizontal and the statistical significance on the vertical axis. The panels C, D, and E in Fig. 4 all use the same scale to allow for comparison. Figure 4C shows transcripts that were differentially regulated within BCBL-1 cells at *P* ≤ 0.001, the top level of the hierarchical cluster. There were 16 transcripts. Their degree of difference was not as great as that between cell lines (compare the scale on both the horizontal axis depicting fold change and the vertical axis depicting the significance level). A complete list of differentially regulated cellular genes is provided in Table S1. Nevertheless, these transcripts pointed to a common biological pathway: PCNA, TUBA1B, TYMS, UBE2T, FEN1, TK1, DUT, STMN1, MCM7, H2AFZ, HMGB2, GMNN, CKS1B, KIAA0101, CKS2, and RPM2. Most of these are cell cycle-regulated genes, in particular genes that are involved in nucleotide synthesis and DNA replication. Many more cell cycle-regulated genes differed between the top two clusters but individually did exceed our significance threshold. Figure 4D shows the next (left-level) cluster split out cells in mitosis as ascertained by the significantly regulated genes CCNB1, CKS2, PLK1, CDC20, TPX2, CCNB2, and NUSAP1. Figure 4E, the right-level cluster split, shows no other genes were differentially regulated within BCBL-1 populations. Specific Gene Ontology pathways for significantly differentially expressed genes of Fig. 4B to D can be seen in Fig. S3. This result suggests that BCBL-1 cells grown under optimal conditions in suspension represent a homogenous population with regard to cellular gene transcription.

10.1128/mbio.03473-21.4 FIG S3 Biological Gene Ontology pathways of the most significant genes from the differential expression experiment featured in Fig. 4. (A) Corresponds to genes indicated in red in Fig. 4B. (B) Corresponds to the genes indicated in red in Fig. 4C. (C) Corresponds to the genes indicated in red in Fig. 4D. Download FIG S3, TIF file, 1.3 MB. Copyright © 2022 Landis et al. 2022 Landis et al. https://creativecommons.org/licenses/by/4.0/ This content is distributed under the terms of the Creative Commons Attribution 4.0 International license.

In every culture, some BCBL-1 cells adhere to the plastic wall (Fig. 1A and B and Movie S1). This was not the phenotype of a particular subclone of BCBL-1 cells but appeared repeatedly at each new passage. It is specific for BCBL-1 cells and was not observed for JSC-1 cells. To enrich for this population, BCBL-1 cells were cultured horizontally on fibronectin-coated flasks in the presence of 5% methyl cellulose. These experiments, however, should be considered preliminary, as they were limited by the purity of the population. No one cellular gene could be identified as a biomarker for the adherent state of BCBL-1 cells. This result supports the notion that even though BCBL-1 cells in culture can adopt two different phenotypes, one representing suspension growth as found in pleural effusions and the other representing tumor cells adhering to the body cavity walls, or even mesenchymal differentiation (75), these phenotypes are likely regulated at the posttranscriptional level.

No one cellular gene emerged as a biomarker that correlated significantly with the different clusters of viral transcription or any one viral gene in BCBL-1 cells. This suggests that, under steady-state conditions, the different patterns of viral transcription represent responses to posttranscriptional signals or even stochastic fluctuations. It may also highlight the limitations of scRNAseq, which only measures the most abundant mRNAs. It is important to also keep in mind that SigClust conducts explicit hypothesis testing to determine how big the distance between any two clusters must be to reach a specific significance cutoff (76). It represents a conservative approach to hierarchical clustering. It is quite possible that, if one had induced massive changes in host and viral transcription, e.g., via HDAC inhibitors or TLR activation across the entire culture (6, 77), correlations between host and viral genes would have emerged. This, however, was not the purpose of these experiments.

## DISCUSSION

KSHV establishes latency in B cells (50, 78–82) and KSHV latent genes, including the viral miRNAs that modify the behavior of developing B cells (83–85). Consequently, KSHV has been associated with a varied spectrum of B cell abnormalities, such as localized hyperproliferation, multicentric Castleman’s disease (16, 66, 67), microlymphoma (86), and solid lymph node-associated (70, 71) as well as body cavity-based PEL (87, 88), KSHV immune reconstitution syndrome (KS-IRIS; and the presumed B cell activation as measured by B cell inflammatory cytokines, such as IL-6), and KSHV-associated inflammatory syndrome (KICS) (89). Different PEL and PEL cell lines both have common and distinct cellular gene transcription profiles (90, 91). The novel insight of this study is that even within clonally selected, long-term *ex vivo*-propagated PEL cell lines, distinct subpopulations of cells exist.

By applying scRNAseq to herpesvirus latency in PEL, we uncovered subpopulations among PEL that differed from each other in terms of viral and cellular gene expression.

(i) The latency locus-associated transcripts were present in the majority of cells, as was the Nut-1/Pan/T1.1 lncRNA. This was consistent with prior bulk transcriptional profiling studies in PEL (24, 31). The vIRF-3 and, to a lesser degree, vIRF-1, -2, and -4 transcripts were detectable in many cells, which was consistent with prior studies (92–94). Transcripts traversing the ORF K15 locus were also consistently detected. Thus, the KSHV latency status is tightly regulated in healthy PEL cells and dominated by the known latent genes, which can be considered essential for PEL survival and potential targets of therapy, including therapeutic vaccination.
(ii) There are limitations to this interpretation, since mRNA levels are dependent on both ongoing transcription and mRNA stability. Typically, scRNAseq experiments are heavily biased towards abundant RNAs. This is of importance for interpreting the Nut-1/T.1.1/PAN results. Whereas the half-life of protein mRNAs is measured in minutes, long noncoding RNAs (lncRNAs) are much more stable. Nut-1/T.1.1/PAN is the most abundant KSHV lncRNA. It is polyadenylated but nuclear localized (26, 51, 95). Nut-1/T.1.1/PAN is also tightly associated with DNA and proteins (95–97). By comparison, the LANA mRNA, which is abundant but, unlike the LANA protein, not particularly stable, was not as consistently detectable as the Nut-1/T.1.1/PAN lncRNA. In the field, Nut-1/T.1.1/PAN lncRNA would be a better biomarker for KSHV-infected cells than LANA mRNA.
(iii) The transcription pattern of the viral IL-6 homolog ORF K2/vIL6 was notable. It was detectable in many cells of the JSC-1 cell line but only a limited number of cells in the BCBL-1 cell line. These single-cell data mirror the biology of ORF K2/vIL6, which exhibits highly variable expression across different KSHV diseases and has multiple modes of transcriptional activation, some that are KSHV RTA dependent and some that are independent (56, 66, 98–101). There are limitations to this interpretation, as K2/VIL6 is coterminal and overlaps ORF2 (102). These scRNAseq data are strongly biased towards the 3′ end of each transcript, as it uses a poly(dT) primer in the first step. They do not capture full-length mRNAs. Hence, it was not possible to ascertain whether the count data reflect ORF 2 or ORF K2/vIL6 message.
(iv) As expected, the two different cell lines JSC1 and BCBL1 differed from each other more so than different batches of the same PEL cell line or subpopulations that emerged within the BCBL-1 cell line. Most of these differences manifested themselves in differences in cellular gene expression, reflecting the unique patient origin and mutational profile of each PEL cell line. This suggests that host cell transcription patterns, rather than viral genes, are important determinants of PEL tumor progression and therapy responses.
(v) The transcription profiles of different batches of the same PEL cell line were similar over time. Batch effects could be removed using a linear model (103, 104). After correction for technical variability, the transcriptionally defined subpopulations within the cell line were stable over subsequent passages, suggesting that they are generated anew at each generation with a fixed frequency. Such a mechanism is consistent with the general partition model for extrachromosomal herpesvirus genomes, which naturally creates transient imbalances in viral gene expression after individual cell division events even if the population as a whole exhibits stable genome partitioning behavior (13, 17, 72). In sum, both clonal mutational events (genetic drift) as well as individual transcription patters contribute to the variability of each cell line.
(vi) At any given time, a fraction of cells in PEL cell lines undergoes lytic reactivation, as measured by KSHV protein expression, e.g., ORF K2/vIL6 or ORF K8.1. Spontaneous reactivation events are rare but result in constitutive low-level shedding of infectious particles into the culture supernatant (28, 37). The experiments reported here were designed to limit spontaneous reactivation events as much as possible by maintaining the cells under ideal culture conditions. In addition, FACS eliminated cells with aberrant cellular transcription, indicative of cell death (105–107), including apoptosis induced in response to KSHV replication. Under these conditions, most late-lytic mRNAs (covering ∼50% of annotated genome) were not detectable at all, suggesting that fewer than ∼1/600 healthy PEL cells at any one time spontaneously enter the lytic phase or that KSHV late transcription is associated with rapid apoptosis and massive changes in overall host transcript levels. The latter hypothesis is consistent with the reported RNA degradation functions of KSHV ORF 37/SOX (108, 109).
(vii) Many more cells transcribed early viral mRNAs (ORF50/Rta, ORF 49, ORF 45, ORF 48, ORF K5, ORF K4, and ORF K8.1) than late, ganciclovir-sensitive viral mRNAs. This suggests that one reason that the early ORF 45 and ORF K8.1 proteins are consistent markers for KSHV reactivation is that they are readily transcribed even if none of the replication-dependent, late genes are expressed (110). Even though ORF 50/Rta was transcribed in some cells, this did not correlate with complete viral reactivation. This suggests that additional safeguard mechanisms are in place to prevent widespread virus transcription in the case of Rta “blips.” One such candidate mechanism is inhibitory DNA and histone modifications, which are well documented in KSHV-infected cells (1, 2, 4, 6). Other candidate mechanisms include the need for cooperating viral immediate-early genes and/or cellular transcription factors.

The analysis of scRNAseq data is far from routine. Some of the limitations have been alluded to above, while others are more theoretical in nature. For instance, it is difficult to define the minimal cluster size of a single-cell experiment. Are single cells also single clusters? How many similar cells does it take to call a cluster, and how like each other do the transcription profiles of the cells within a cluster have to be? The SigClust algorithm addresses those questions explicitly (76). In flow cytometry, a common conservative estimate uses ≥5% of all events to define a new cluster (107). The five clusters defined in Fig. 3 fall above this threshold and have ≥70 cells each.

A recent report advocates for a mesenchymal origin for PEL (75). The authors noted that in effusions of HIV^+^ PEL patients, a population of adherent mesenchymal cells existed, which, with time, gave rise to B1 lymphoid cells. Whereas the adherent mesenchymal cells were KSHV negative, the emerging B1 lymphoid cells in some of the cultures were KSHV positive. Similar claims have been advanced the postulate of a mesenchymal origin of KS (111–117). BCBL-1 cells, but not JSC-1 cells, had an innate ability to adhere to the plastic side of the culture flask, which could be enhanced by fibronectin coating and reducing turbulence. Unfortunately, this crude method of culture was not able to obtain a pure enough population to yield a statistically different cellular or viral gene expression signature. Whether this attachment phenotype reflects a cell cycle state or is due to posttranscriptional regulatory events alone remains an open question. These studies underscore the plasticity of KSHV-infected cells by identifying five clusters of viral transcription. They are consistent with the general impression that KSHV driving endothelial reprogramming (blood endothelial to lymphatic endothelial, or BEC to LEC) as well as endothelial-to-mesenchymal transition (EMT) but should not be construed as favoring the same idea for PEL. Further studies are needed. Understanding the population composition of KSHV-associated malignancies at the single-cell level represents the next step, can be expected to yield important insights into the fundamental biology of the virus, and may uncover new avenues of intervention.

## MATERIALS AND METHODS

### Tissue culture.

Cells were obtained from the American Type Tissue Culture Collection (ATCC) or the NIH AIDS reagent program. Cells were cultured in RPMI 1640 (Gibco) supplemented with 100 U/ml penicillin-streptomycin (Gibco), 2 mM l-glutamine (Gibco), and 10% Fetalgro bovine serum (VWR). Cells were maintained at 37°C in 5% CO_2_ and passaged for no more than 3 months at a time. Specifically, cultures were seeded at 0.25 × 10^6^ cells/ml and used at 48 h after culture initiation or diluted 1:5 at 72 h for routine passage. Cells were imaged continuously on a LEICA PAULA smart cell imager to quantitate growth and density. Cell identity was confirmed by STR typing (PowerPlex 16HS assay; Promega). All cells underwent periodical mycoplasma testing (LT07-701; Lonza).

For adherent cell enrichment, a fibronectin-coated 25-cm^2^ plate was inoculated with cells in 10 ml of 50% culture medium (0.5 × 10^6^ cells)–5% methylcellulose to facilitate adherence. After 48 h, regular RPMI replaced the spent methylcellulose-containing medium, and the cells were maintained until the day of scRNAseq (the time lapse recording was on adherent cells cultured without methylcellulose). At that time, the culture medium was discarded and the adherent cells washed twice with phosphate-buffered saline (PBS) without magnesium (14190-144; Gibco). Next, the cells were incubated in trypsin-PBS for 10 min at 37°C and pelleted by low-speed centrifugation. The cell pellet was suspended in 1,000 μl of Hanks’ balanced salt solution with 2% fetal bovine serum, and the cells were passed through 35-μm cell strainers (352235; Corning Falcon). The cells were sorted directly into prepared Precise WTA single-cell encoding plates (634100; BD) on 96-well plates using a Becton, Dickinson FACSAria II while leaving four wells empty for quality control per the manufacturer’s protocol. The manufacturer’s protocol settings ensure that at most 1 cell per well is deposited. For plate 8, two cells were sorted into each well.

### Single-cell RNA sequencing.

These studies used the Precise whole-transcriptome analysis single-cell kit (634100; BD) by following the manufacturer’s protocol. The pooled library was submitted to GenWiz Inc. (Plainfield, NJ) for Illumina HiSeq sequencing using 2 × 250 bp paired reads at 3 pools per lane with 15% Phi-X spike as a control. The median ± standard error of the mean per library was 6 × 10^7^ ± 5 × 10^6^ reads, 1 × 10^6^ ± 2 × 10^5^ unique molecular identifiers (UMI), and 11,847 ± 296 genes. Note that libraries 5 and 6 were sequenced twice on two different HiSeq lanes.

For mapping the scRNA data to the human genome, the manufacturer-supplied analysis pipeline (Precise whole transcriptome assay analysis pipeline v2.0; BD) was used as implemented at https://www.sevenbridges.com/. Rather than using total counts per ORF, the BD methodology calculates unique molecular identifiers (UMI), which reduces reverse transcription-PCR bias (105, 118). The manufacturer-supplied analysis pipeline yielded the UMI data matrix for each human gene in each cell for each experiment, termed data set 1.

To map the scRNA reads to the KSHV genome, a methodology similar to that for the human genome was used. This allowed for consistent comparison of UMI counts between reads mapping to the human genome versus the KSHV genome.

Prior to analysis, the cells were subjected to a series of quality control measures and filtering steps based on human gene transcription. The code and data are available on the paper bitbucket repository. The following are the filtering conditions used in the context of 20,240 protein-coding genes and 1,152 cells. Cells were required to have greater than 5,000 total UMI and greater than 1,500 uniquely detected cellular genes. If a cell did not meet both requirements, then the cell was considered apoptotic or a technical failure and removed. For filtering on the human genes, the following conditions needed to be met: the gene was expressed in greater than 0.5% of all cells, was a protein-coding gene, and was not related to mitochondrial or ribosomal expression. If any of the previous conditions was not met, then that gene was excluded from the analysis (see Fig. S1 in the supplemental material). Other sophisticated filtering steps were explored, e.g., see reference 105, but upon experimentation, the above-described criteria and thresholds emerged as the most reliable and biologically coherent across this data set and experimental design. This analysis pipeline yielded data set 2.

10.1128/mbio.03473-21.2 FIG S1 Cellular gene thresholding visualization. Panel A represents a histogram of total UMI counts for all 1,152 cells. Panel C represents the total UMI counts, except each cell is ranked by its UMI count depth. Panel B represents a histogram of the number of detected genes per cell. Panel D is a scatter plot of the total UMI counts per cell and the number of genes detected per cell. A red line is present in panels A, C, and D, indicating the 5,000-UMI count threshold. A red line is present in panels B and D representing the 1,500 thresholds for detected genes per cell. Download FIG S1, TIF file, 0.7 MB. Copyright © 2022 Landis et al. 2022 Landis et al. https://creativecommons.org/licenses/by/4.0/ This content is distributed under the terms of the Creative Commons Attribution 4.0 International license.

### scRNAseq virus data analysis.

The virus scRNAseq analyses were subjected to additional normalization: (i) as many KSHV genes are 3′ coterminal, it was useful to combine the counts for the multiple genes in the KSHV latency locus, comprised of K12, ORF71, ORF72, and ORF73; (ii) the KSHV miRNA genes are removed, since this scRNAseq method did not capture miRNAs, pre-miRNAs, or any RNAs that are not polyadenylated (19). The data were further processed by providing easily recognizable labels for the plates and by changing some of the names for the viral genes, from the nomenclature used in the reference sequence NC_009333 to their more biologically intuitive names that have become the *de facto* standard in the field. The viral genes were log normalized in a manner similar to that for the human genes, except they were scaled by the size factors of the human cellular genes, since the viral gene population is low expressing. For heatmap representations, counts were scaled to yield a binary signal, as this aided visualization; however, all computations were performed on the transformed count data. After these processing steps, 70,644 data points, comprising 841 individual cells, were available for heatmap visualization. Unsupervised clustering was performed using either the k-means algorithm (1,000 iterations from 25 random starting points) or hierarchical clustering based on Euclidian distance using Ward’s method as implemented in R v3.6.2. Code and documentation are available at the bitbucket repository for this work: https://bitbucket.org/dittmerlab/scrnaseq_bcbl1/src/master/.

### scRNAseq cellular data analysis.

UMI were obtained, and cells and genes were filtered on raw UMI counts as described above for QC. The final data set contained 841 cells and 12,908 genes for a total of 10,855,628 data points. We lost 27% of our cells upon filtering and upwards of 36% of the genes. Most genes were lost due to low gene frequency, i.e., the gene was only detected in a few cells at very low levels. UMI was normalized by computing the log-normalized expression values. This method was utilized through the R package scran. Log-normalized counts have greater stability and decrease the variance between libraries.

Typically, scRNAseq data show more variation than bulk RNAseq data (68, 119). They tend to be zero-inflated and overdispersed. For experiments combining multiple libraries and/or biological samples batch effects are significant, and the data need to be adjusted for it; multiple methods have been proposed (69, 120–122). We used analysis of variance (ANOVA) on well-normalized, transformed UMI to arrive at a single correction coefficient per plate batch for nonzero entries. This model was as effective as other more complicated models, e.g., Seurat (123), perhaps since each batch did not contain mixtures of cells in various proportions to each other but only cells of a clonal cell line.

The batch and library size-adjusted data were subjected to hierarchical clustering with FDR adjustment using Ward-D as the method and a Euclidian distance matrix as implemented in the SigClust package (76).

First, the libraries from biological replicates of plate 5 were split across two different Illumina sequencing lanes each. A pairwise comparison of transformed UMI across all cells and genes generated an estimate of the variation that was introduced by the sequencing step alone. The two libraries were highly correlated with a regression coefficient of *r*^2^ = 0.997 (Fig. S2A). The perfectly horizontal distribution on the MA plot (103) indicated that variation of normalized UMI counts was constant across the range of expression levels in the experiment (Fig. S2E). This attests to the high reproducibility of this technology. Second, two cells rather than one cell per well were sorted into plate 8 to explore sensitivity and linear range of the cDNA conversion step. Gene expression levels for the plate containing two cells per well correlated linearly with the plate containing only one cell per well with *r*^2^ = 0.961 (Fig. S2B and F). This demonstrates that reagents were not limiting the cDNA reaction. A few genes were present at significantly higher counts (red and orange dots in Fig. S2B) in the 2 cells/well plate than in the 1 cell/well plate, suggesting that providing twice as much input RNA improved the cDNA conversion rate for weakly transcribed genes. Overall variability for these different library preparations was increased (Fig. S2E versus F). This was expected, as two different library preparations were compared, not two different sequencing runs on the same library. Third, two different PEL cell lines were compared, BCBL-1 and JSC-1 (37, 38). The cell lines differed dramatically regarding cellular gene expression (Fig. S2C and D). This comparison served as a positive control and to justify the level of the FDR. Any transcript that exhibited a level of variation of *M* ≥ 2 (red dots in Fig. S2G) was considered differentially regulated between the two different cell lines. Extending this argument to different clusters of the same cell line, any transcript within a population of BCBL-1 cells was considered differentially regulated between single cells. Conversely, biological replicates for BCBL-1 cells served as a negative control (Fig. S2D and H). BCBL-1 cells were analyzed at three different dates (1 week apart). Plates 1 and 2 were FACS sorted on two separate dates, 1 week apart, and each was processed into a different library. Plates 3 to 5 were sorted on a different, third date, and each was processed into a different library. As expected, the biological variation between different calendar dates was larger than variation of different libraries made on the same calendar date, but not by much (Fig. S2D and H compared to Fig. S2A and E). Together, these measurements established biological plausibility for differential gene expression beyond statistical significance cutoffs alone. If the difference in mRNA transcription patterns between any two cells was less than that across biological replicates, it is unlikely that the two cells represent different subpopulations. If the difference in mRNA transcription patterns between two cells in the same biological replicate approached the level of difference between all BCBL-1 and all JSC-1 cells, it reflected biologically relevant differences between these two cells.

As scRNAseq is prone to catastrophic failures affecting only a single cell, the data sets expectedly contained outliers. These outliers could represent wells (i) that did not receive any cell during the initial FACS procedure, (ii) that received a cell in the process of cell death, where mRNA was degraded, (iii) where mRNA extraction and reverse transcription failed, and (iv) where pipetting failed. These types of errors affect steps before library construction. They are expected to affect the gene expression profile globally, as opposed to specific changes in groups of genes, as would be observed for physiologically significant differences. These outliers were removed prior to analysis (see Materials and Methods). Afterwards, UMI count data were adjusted to remove batch by library size normalization and transformed to stabilize variation using the algorithm of the scran R package. All further analyses were conducted on adjusted data.
