# Supplementary figures and images for: Evidence for Multiple Subpopulations of Herpesvirus-Latently Infected Cells
Source: mBio. 2022 Jan 4;13(1):e03473-21. doi: 10.1128/mbio.03473-21 (PMC8725583; doi:10.1128/mbio.03473-21)

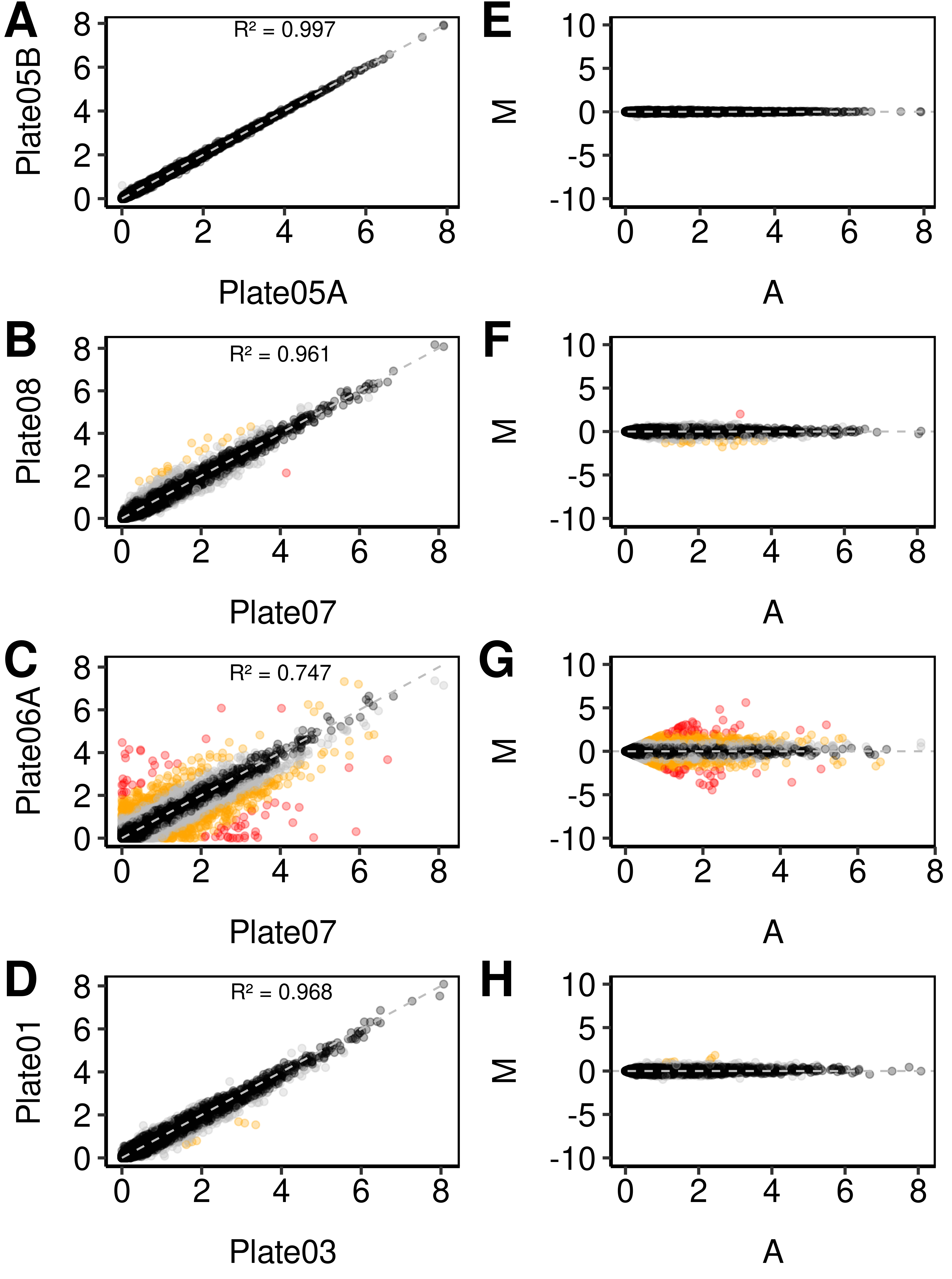

Supplement: FIG S2 [file mbio.03473-21-sf002.tif]

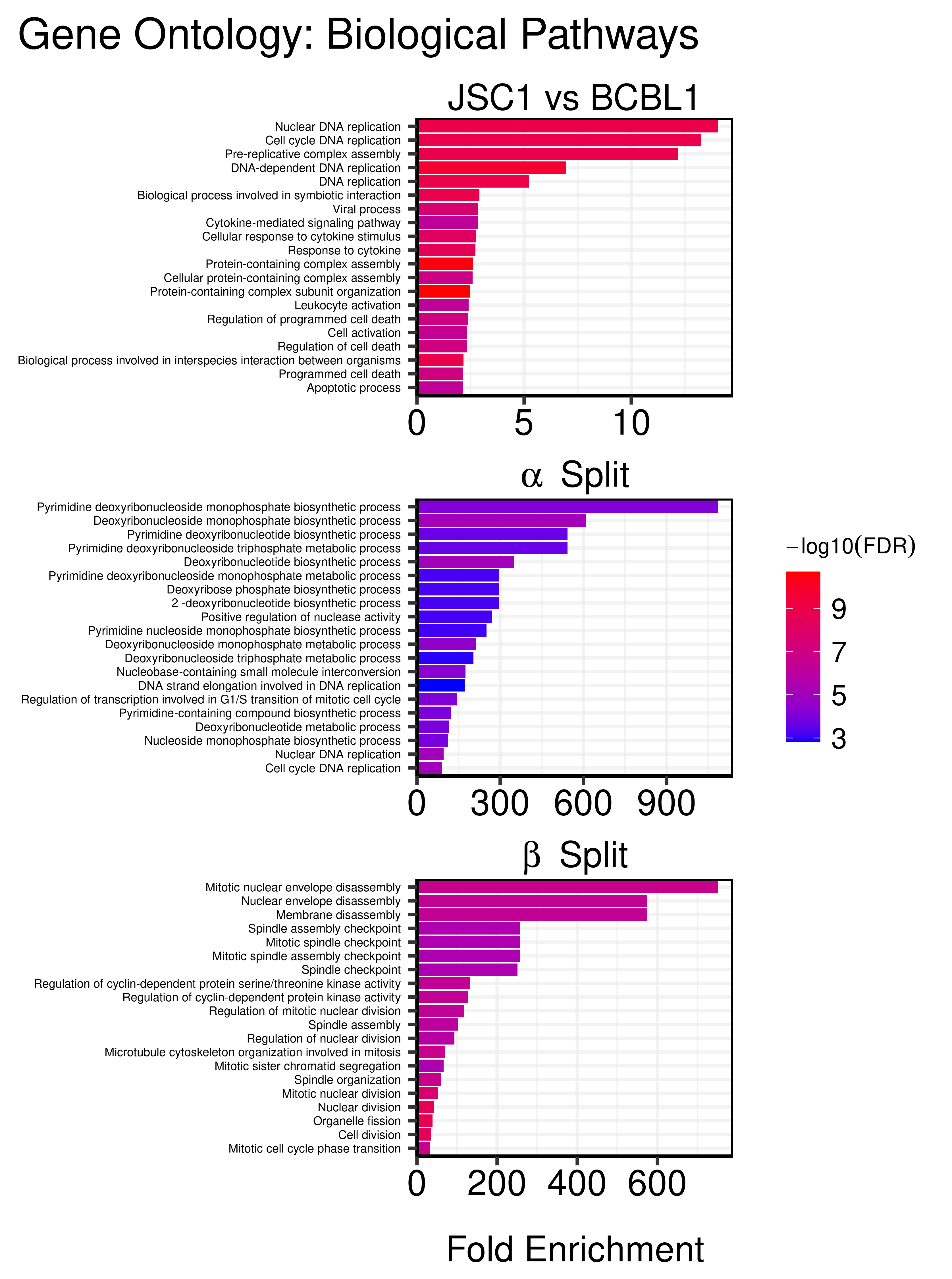

Supplement: FIG S3 [file mbio.03473-21-sf003.tif]

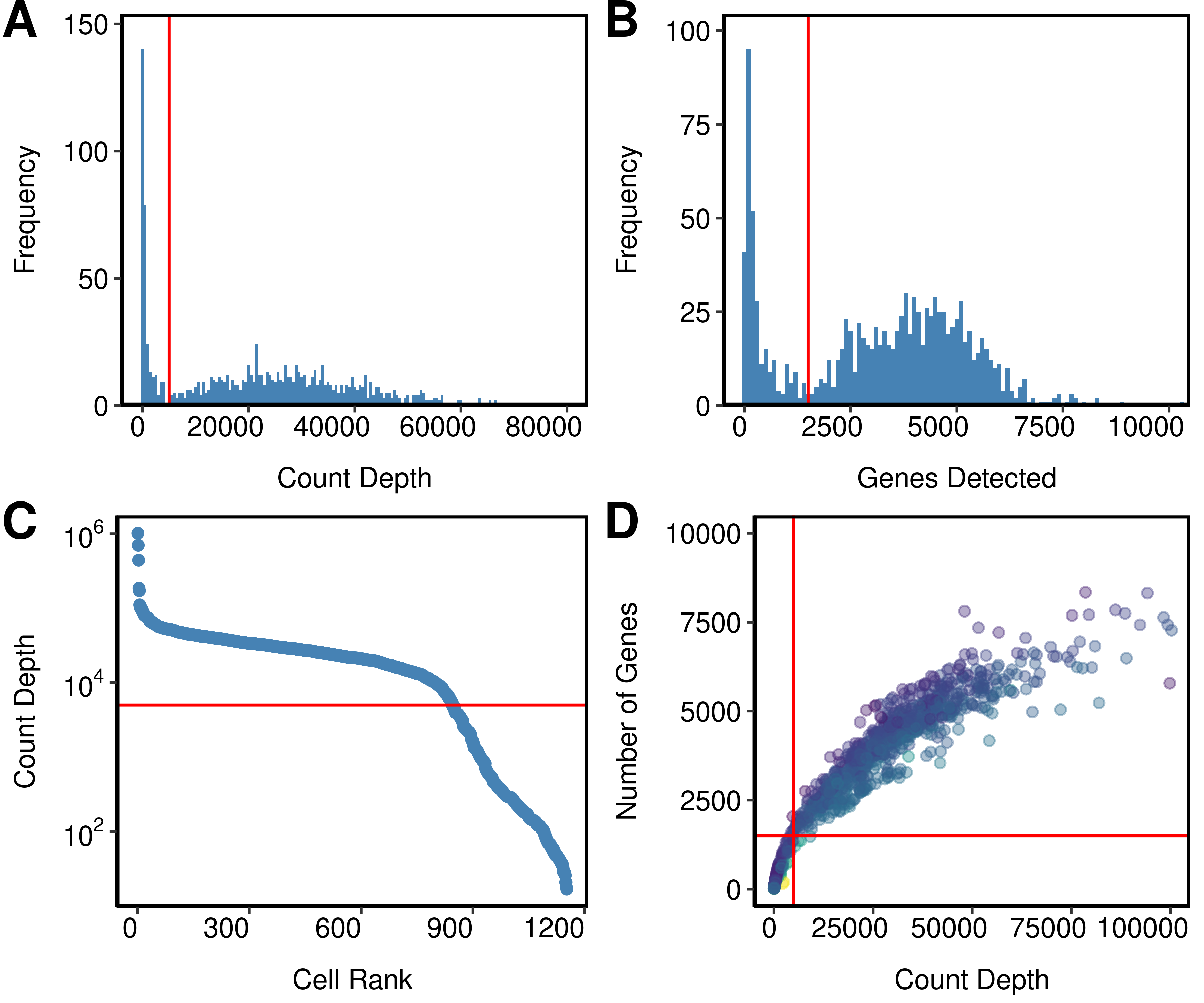

Supplement: FIG S1 [file mbio.03473-21-sf001.tif]
